# Clinical outcomes of bilateral implantation of new generation monofocal IOL enhanced for intermediate distance and conventional monofocal IOL in a Korean population

**DOI:** 10.1186/s12886-023-02897-2

**Published:** 2023-04-17

**Authors:** Wan Kyu Choi, Hyo Ji Han, Hyeck-Soo Son, Ramin Khoramnia, Gerd U. Auffarth, Chul Young Choi

**Affiliations:** 1 Department of Ophthalmology, Kangbuk Samsung Hospital, Sungkyunkwan University School of Medicine, 29 Saemunan-Ro, Jongno-Gu, Seoul, 03181 Republic of Korea; 2 Department of Ophthalmology, The David J. Apple International Laboratory for Ocular Pathology, University of Heidelberg, Im Neuenheimer Feld 400, 69120 Heidelberg, Germany

**Keywords:** Eyhance, Tecnis, Monofocal IOL, Cataract

## Abstract

**Background:**

To compare the clinical outcomes of bilateral implantation of enhanced intermediate function intraocular lenses (IOLs) and standard monofocal IOLs.

**Methods:**

In this prospective, randomized, comparative controlled study, we compared the visual outcomes of patients who underwent bilateral cataract surgery at the Kangbuk Samsung Hospital, Sungkyunkwan University School of Medicine, with either enhanced monofocal IOLs (Tecnis Eyhance, ICB00, Johnson and Johnson Vision Care, Inc) (Group 1) or standard monofocal IOLs (Tecnis, ZCB00, Johnson and Johnson Vision Care, Inc) (Group 2). The assessment included monocular and binocular uncorrected distance visual acuity (UDVA), uncorrected intermediate (UIVA at 60 cm) and near (UNVA at 40 cm) visual acuity, uncorrected defocus curves, contrast sensitivity testing (CST), and reading speed test using Quality of vision was evaluated using the Visual Function Questionnaire (VFQ-25).

**Results:**

At 3-months postoperatively, monocular and binocular outcomes of UIVA and UNVA were statistically significantly better in Group 1 (*P* < 0.05). The binocular uncorrected defocus curve of Group 1 showed statistically significantly better outcomes at vergence ranges of -1.5 to -4.0 D (*P* < 0.05). Significantly higher reading speed test was also observed in Group 1 in all ranges tested (1.0 to 0.1 LogMAR) (*P* < 0.05). There were no statistically significant differences in CST between groups.

**Conclusions:**

Bilateral implantation of enhanced monofocal IOLs provided better vision at intermediate and near distances compared to standard monofocal IOLs, while maintaining good distance vision and contrast sensitivity.

## Background

Today, multifocal intraocular lenses (IOLs) are widely used to provide good spectacle independent vision at far, intermediate, and near distances [1]. However, patients with such multifocal optics may experience photic phenomena such as glare and halo or perceive reduced contrast sensitivity [2]. Thus, various studies have sought to investigate whether combining trifocal, bifocal, extended depth of focus (EDoF), and monofocal IOLs may minimize such optical side effects while maintaining good visual quality [3, 4].

Previously, we performed a combined implantation of trifocal and EDoF IOLs in cataract patients and observed good visual outcomes in far to near distances as well as a high spectacle independence [5]. In another study, we compared the visual function in patients who underwent either blended implantation of bifocal and EDoF IOLs or bilateral implantation of trifocal IOLs, and we reported satisfactory spectacle-independent vision at far, intermediate, and near distances in both groups [6]. In all three strategies we investigated, however, there still remained the issue of photic phenomena that caused some degree of patient dissatisfaction.

The Tecnis® Eyhance (Model ICB00, Johnson and Johnson Vision Care, Inc) is a recently developed monofocal IOL with a higher-order aspheric anterior surface designed to improve intermediate vision. As it has been shown to provide good uncorrected visual quality at far to intermediate distances, the Tecnis Eyhance is increasingly gaining popularity [7–11]. Kang et al. reported that Tecnis Eyhance can not only achieve superior intermediate vision and comparable visual performance at distance, but also generate low incidence of photic phenomena comparable to that of a Tecnis standard monofocal IOL [10]. Similar results were observed in other studies that compared the Tecnis Eyhance with Alcon and Rayner’s standard monofocal IOLs [12, 13].

In this study, we sought to compare the visual outcomes, contrast sensitivity, reading speed, photic phenomena, and patient satisfaction in patients who underwent binocular implantation of Tecnis Eyhance IOL (Model ICB00) and binocular implantation of a standard Tecnis monofocal IOL (Model ZCB00) that shares the same optical platform.

## Methods

This prospective, randomized, and comparative controlled study included patients with age-related cataracts who underwent bilateral cataract extraction with phacoemulsification and IOL implantation with either an enhanced monofocal IOL (Tecnis Eyhance, ICB00, Johnson and Johnson Vision Care, Inc) (Group 1) or a standard monofocal IOL (Tecnis, ZCB00, Johnson and Johnson Vision Care, Inc) (Group 2). Both groups comprised of 25 patients and the diagnosis was made using the lens opacities classification system (LOCS) III.

This study followed the tenets of the Declaration of Helsinki and was approved by the Institutional Review Board of Kangbuk Samsung Hospital (IRB File No. 2019–12-030). All study participants signed the informed consent before enrollment.

Inclusion criteria were patients aged 50 years or older at time of study enrollment, who underwent surgery of fellow eye 1 week after surgery of the first eye, showed postoperative visual potential of 20/25 or better, and had pre-existing corneal astigmatism of less than 1.25 D. Exclusion criteria were: any general condition that may affect the surgical outcomes and a history of ocular trauma, surgery, or pathology that may limit the visual function.

All patients received preoperative ophthalmic examination including mesopic (3 cd/${m}^{2}$) pupillometry, manifest refraction, uncorrected (UDVA) and corrected distance visual acuity (CDVA), uncorrected intermediated (UIVA) and near visual acuity (UNVA), topography (Galilei G6: Ziemer Ophthalmic Systems AG), corneal aberration (KR-1W Wavefront Analyzer; Topcon Europe Medical BV), optical biometry and keratometry (IOLMaster 700, Carl Zeiss Meditec), slit-lamp examination and fundoscopy. The mesopic pupil diameter was measured using the length of slit light with minimum width during slit-lamp examination.

All surgeries were performed by one surgeon (CYC): under topical anesthesia, a 2.2 mm corneal incision was made, followed by manual capsulorrhexis and phacoemulsification. All IOLs were implanted in the bag. Postoperative target refraction was determined as the lowest myopic value after emmetropia using the Haigis formula in IOL calculation.

Follow-up examinations were performed at 1-week, 1-month, and 3-months after implantation of the second IOL. Main outcome measures included visual acuities, monocular and binocular defocus curves, contrast sensitivity testing (CST), reading speed test, and patient questionnaires. UDVA, UIVA at 66 cm, and UNVA at 40 cm were measured using the Early Treatment Diabetic Retinopathy Study charts (ETDRS; Vector Vision, Ltd., Greenville, OH, USA). Uncorrected monocular and binocular defocus curves were obtained for distance vision with the ETDRS charts at intervals of 0.50 spherical D from − 4.00 to + 1.00 D. CS was measured at 3.0, 6.0, 12.0, and 18.0 cycles per degree (cpd) under photopic (85 cd/m^2^) and mesopic (3 cd/m^2^) conditions with and without glare with the CSV-1000 (Vector vision, Inc., Greenville, OH, USA).

Patients' subjective satisfaction was analyzed using the quality of vision (QoV) and vision-related quality of life (QoL) questionnaires both preoperatively and 3-month postoperatively, while spectacle independence was assessed using the 25-item National Eye Institute Functional Questionnaire (NEI VFQ-25).

The binocular reading speed at 40 cm was measured at 3-months postoperatively as described by the Korean Reading Speed Application tester [5, 6]. Letter sizes from 0.0 logMAR to 1.0 logMAR were displayed in steps of 0.1 logMAR. Patients were asked to read sentences of different sizes one after the other. Reading speed (words per minute) was automatically calculated by its own system “Hangul”.

Statistical analysis was performed using the SPSS software (Version 24.0: SPSS, Inc). Intergroup comparisons of monocular and binocular visual outcomes were performed with the independent two sample t-test and Pearson’s chi-square test. The Mann–Whitney U test was used to compare quantitative variables (such as refraction) and reading speed. The *t* test for independent samples was used to compare overall satisfaction and spectacle independence. For the adjustment of *P* values, the Bonferroni correction was used. Data were expressed as mean and standard deviation. For all analyses, the level of significance was a *P* value of less than 0.05. Normal distribution of our data was confirmed via histogram.

## Results

Demographic and preoperative clinical characteristics of all patients are shown in Table 1. There were no statistically significant differences in any of the variables between the two groups.

**Table 1:** Demographic and preoperative clinical characteristics of study participants

Variable	Eyhance (Group 1)	Tecnis (Group 2)	*P* value
Age (y)	70.9 ± 5.8	71.8 ± 5.8	0.577
Gender			0.370
Male (%)	10 (40)	7 (28)	
Female (%)	15 (60)	18 (72)	
SE (D) (*n* = 50)	0.28 ± 1.87	0.07 ± 1.77	0.572
Photopic pupil size (mm)	2.3 ± 0.5	2.4 ± 0.4	0.350
Mesopic pupil size (mm)	3.8 ± 0.8	3.9 ± 0.8	0.450
UDVA (logMAR)			
Monocular (*n* = 50)	0.39 ± 0.32	0.36 ± 0.36	0.652
Binocular (*n* = 25)	0.25 ± 0.24	0.22 ± 0.18	0.619
CDVA (logMAR)			
Monocular (*n* = 50)	0.18 ± 0.27	0.22 ± 0.24	0.409
Binocular (*n* = 25)	0.07 ± 0.13	0.13 ± 0.13	0.107
UIVA (logMAR)			
Monocular (*n* = 50)	0.33 ± 0.27	0.26 ± 0.21	0.156
Binocular (*n* = 25)	0.23 ± 0.24	0.37 ± 0.28	0.069
UNVA (logMAR)			
Monocular (*n* = 50)	0.46 ± 0.25	0.47 ± 0.26	0.802
Binocular (*n* = 25)	0.37 ± 0.25	0.38 ± 0.20	0.914

*SE* spherical equivalent, *UDVA* uncorrected distance visual acuity, *CDVA* corrected distance visual acuity, *UIVA* uncorrected intermediate visual acuity, *UNVA* uncorrected near visual acuity, *logMAR* logarithm of the minimal angle of resolution

Table 2 lists the mean binocular and monocular UDVA, CDVA, UIVA, UNVA, and spherical equivalent (SE) values of both groups measured 3-month postoperatively. Group 1 achieved better monocular UDVA as well as monocular and binocular UIVA and UNVA compared to Group 2 (*p* < 0.05 for all cases). Binocular UDVA (*p* = 0.146) and CDVA (*p* = 0.193) were better Group 1 compared to Group 2, but there were no statistically significant differences.

**Table 2:** Post-operative visual acuities and spherical equivalents

Variable	Eyhance (Group 1)	Tecnis (Group 2)	*P* value
UDVA (logMAR)			
Monocular (*n* = 50)	0.03 ± 0.05	0.07 ± 0.09	0.002
Binocular (*n* = 25)	0.01 ± 0.03	0.04 ± 0.07	0.146
CDVA (logMAR)			
Monocular (*n* = 50)	-0.02 ± 0.07	0.02 ± 0.10	0.007
Binocular (*n* = 25)	-0.03 ± 0.06	-0.01 ± 0.08	0.193
UIVA (logMAR)			
Monocular (*n* = 50)	0.05 ± 0.05	0.12 ± 0.13	0.001
Binocular (*n* = 25)	0.04 ± 0.05	0.10 ± 0.14	0.026
UNVA (logMAR)			
Monocular (*n* = 50)	0.20 ± 0.14	0.33 ± 0.14	< 0.001
Binocular (*n* = 25)	0.14 ± 0.13	0.29 ± 0.14	< 0.001
SE (D) (*n* = 50)	-0.47 ± 0.29	-0.45 ± 0.25	0.773

**Fig. 1:**
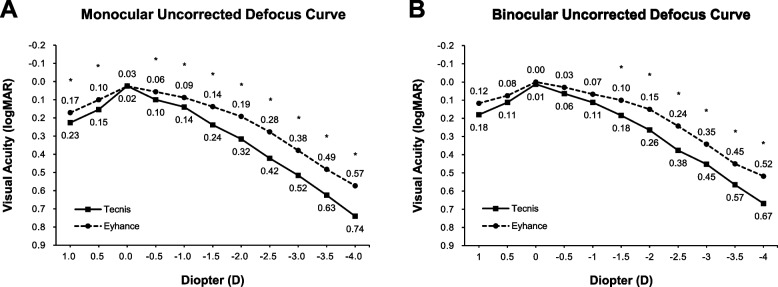
Monocular and binocular uncorrected defocus curves measured at 3-months postoperatively. (* = *p* < 0.05)

Group 1 showed better visual acuity than Group 2 in monocular defocus curve from + 1.0 to -4.0 (*p* < 0.05) except at 0 D (*p* = 0.948). In binocular defocus curve testing, Group 1 demonstrated statistically significantly superior visual acuities from -1.5 to -4.0 D (*p* < 0.05) (Fig. 1).

**Fig. 2:**
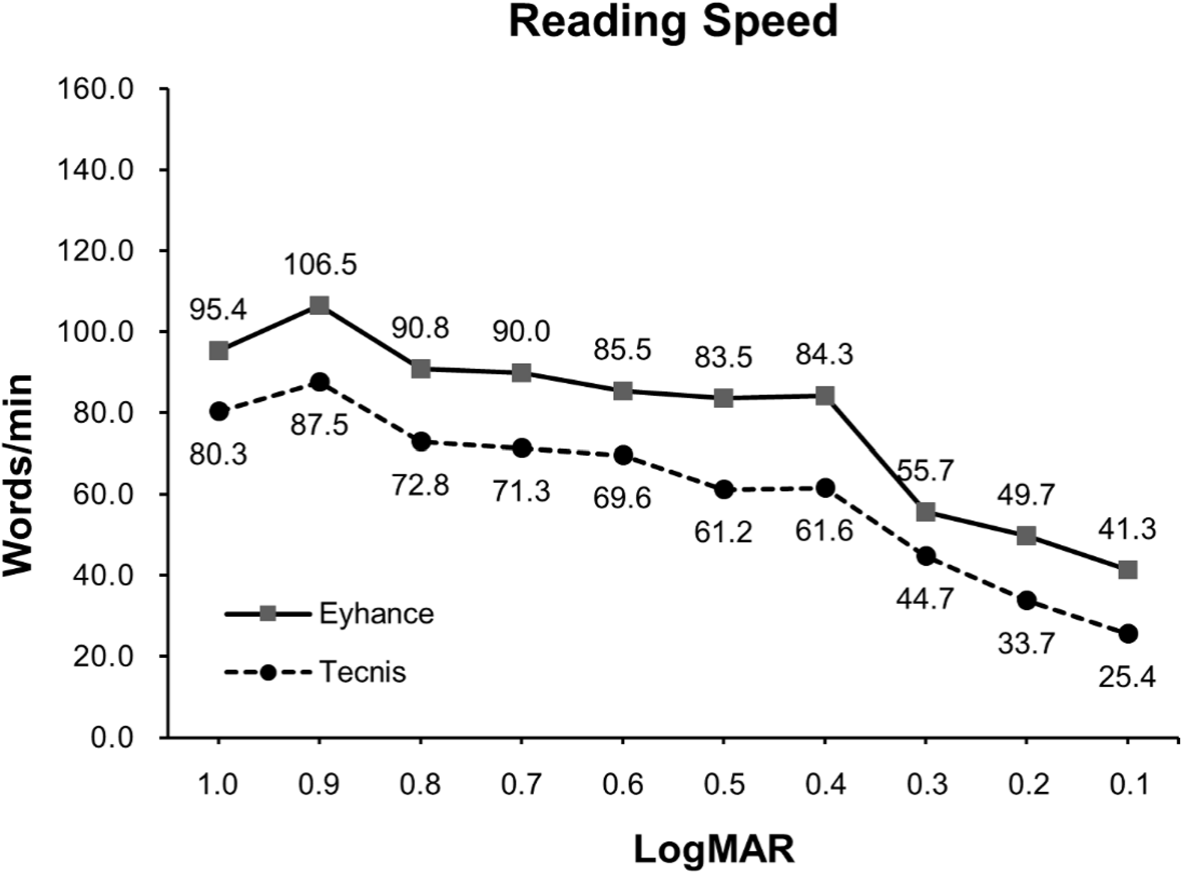
Reading speed measurement performed at 40 cm at 3-months postoperatively

As shown in Fig. 2, Group 1 showed statistically significantly better binocular reading speed than Group 2 for all letter sizes (*p* < 0.05) except for 0.5 logMAR (*p* = 0.223).

**Fig. 3:**
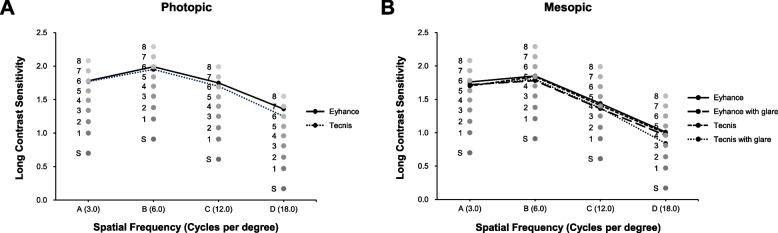
Contrast sensitivity testing at 3-months postoperatively

Figure 3 demonstrates postoperative monocular and binocular contrast sensitivity results under photopic and mesopic conditions with and without glare. For all light conditions, no statistically significant differences were observed at any spatial frequency between the two groups.

**Fig. 4:**
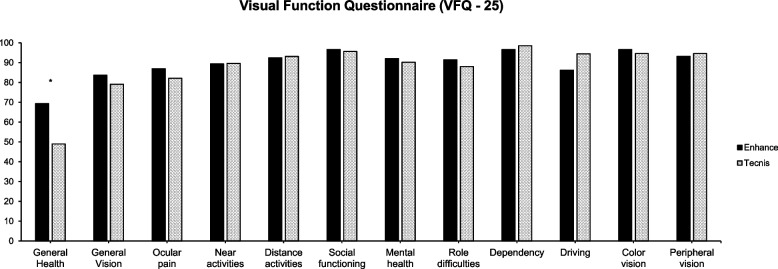
Post-operative 3 month visual function questionnaire (VFQ-25) (* = *p* < 0.05)

The results of the VFQ-25 questionnaire are shown in Fig. 4. All patients reported better outcomes in every category except for ocular pain compared to preoperative values, although there were no statistically significant difference except in general health between Group 1 and Group 2 postoperatively.

**Fig. 5:**
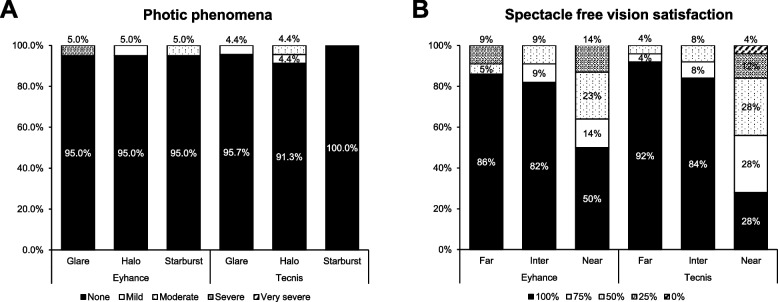
Questionnaire results for photic phenomena and spectacle independent vision satisfaction at 3-months postoperatively

Questionnaire results regarding the perception of photic phenomena and spectacle independent vision satisfaction are shown in Fig. 5. In both groups, more than 90% of patients reported to not experience any glare, halo or starburst phenomena, still there were no statistically significant differences between two groups. Group 1 showed higher spectacle independent vision satisfaction at near distance than Group 1, while there were no statistically significant difference in vision satisfaction at any distances.

**Fig. 6:**
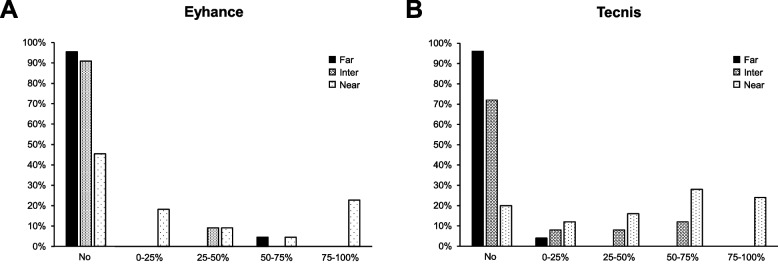
Questionnaire outcomes for spectacle independence for Groups 1 (**A**) and 2 (**B**) assessed at 3-months postoperatively

Both groups showed very high rate of spectacle independence for far distance with over 90% of patients in each group requiring no glasses, while the rate of spectacle independence at intermediate distance was higher in Group 1 (91%) compared to Group 2 (72% (Fig. 6).

## Discussion

In this study, we compared the clinical outcomes after bilateral implantation of an enhanced monofocal IOL and a standard monofocal IOL to investigate the efficacy of the novel optical design for intermediate distance. At 3-months postoperatively, patients with bilateral Tecnis Eyhance IOL demonstrated statistically significantly better monocular and binocular UIVA and UNVA in addition to superior monocular UDVA and CDVA compared to patients with bilateral standard monofocal IOLs. Most importantly, the incidence of dysphotopsia perceived by patients with bilateral Eyhance IOLs was comparable to that of patients with bilateral standard monofocal lenses, demonstrating the efficacy of the Eyhance IOL in providing good vision at all distances while minimizing the optical side effects.

Our findings are in alignment with those of previous reports which demonstrated superiority of the Eyhance IOL in UDVA and UIVA [7–13]. The slight disparities in the reported visual outcomes between studies may be ascribable to the differences in distances at which the visual acuity was measured (e.g. 66 or 80 cm) and the range in postoperative SE (-0.27 to -0.36 in mean value) [11, 14–17]. Our findings also confirm the results of a previous study which showed that the Tecnis Eyhance IOL leads to higher tolerance for residual refractive error compared to a standard monofocal IOL [14].

Both the monocular and binocular defocus curves measured in Group 1 demonstrated a wider and smoother slope than those of Group 2. While the defocus curves from previous studies [15] demonstrated a higher and sharper peak at 0 SE, it is important to note that we obtained an *uncorrected* defocus curve. Indeed, corrected defocus curves would represent the inherent characteristics of the IOLs themselves better; however, it would not be fully representative of the real-life visual function perceived by patients who now mostly live independent with spectacle. Furthermore, assessing the defocus curve with additional trial lenses to correct the residual refractive error may induce wavefront errors, which may lead to fictitious results.

Reading speed is a better indicator of the patients’ actual visual function in daily life and thus has been widely used in numerous clinical studies to characterize the functional outcomes [18, 19]. In our analysis, patients with bilateral Eyhance IOL implantation showed superior reading speed for all letter sizes and visual ranges tested (1.0 to 0.1 LogMAR) compared to those with a standard monofocal IOL. It is important to note that in this study the reading performance was tested using the Korean alphabet known as ‘Hangul’, which is commonly considered to be more complicated in design compared to the English alphabet, composed of vowel and consonants in particular position lather than just enumerating it in a row. Reading performance in other languages may therefore differ from that of our study. To the best of our knowledge, this is the first report comparing the reading performance of these IOLs in a Korean population.

As reported by previous studies [11, 14–16], the contrast sensitivity was within age-adjusted normal range for both groups and did not show any statistically significant differences under all light conditions tested, which is expected given that monofocal IOLs generally do not deteriorate the contrast sensitivity [20] and the Tecnis Eyhance employs the same optical platform as the Tecnis monofocal IOL examined in this study.

We also evaluated the patients’ subjective satisfaction in their daily life using QoV, QoL, and NEI VFQ-25 questionnaires. Both groups showed improvement postoperatively in all questionnaires and showed excellent outcomes in terms of photic phenomena, with more than 90% of patients indicating to experience ‘none’ of photic phenomena in any situation. As the Tecnis Eyhance features an additional optic design in its central optic to generate intermediate vision, one may expect a higher rate of positive dysphotospia. Our results, however, could not substantiate this hypothesis as we observed a comparable rate of photic phenomena in both groups. Given that more than 90% of patients in Group 1 indicated to be spectacle independent for performing tasks in far and intermediate distances, our results suggest that the Tecnis Eyhance IOL is able to provide satisfactory far and intermediate vision, while causing minimal optical side effects.

Although this study may be limited by its relatively short follow-up period and a small sample size, this is the first prospective, randomized controlled study aimed at characterizing the patients’ daily visual performance after bilateral implantation of the Tecnis Eyhance IOL by assessing various visual parameters including reading performance.

## Conclusions

In conclusion, bilateral implantation of an enhanced monofocal IOL demonstrated very good uncorrected far and intermediate visual acuities compared to a standard monofocal IOL that shares the same optical platform. Spectacle independence was high at far and intermediate distances. As evidenced by the defocus curve, patients with bilateral enhanced monofocal IOLs had strong advantages at intermediate distance. Enhanced monofocal IOLs may therefore present a good option in patients with higher demand for distant and intermediate vision without any concern of positive dysphotopsia.

## Data Availability

The datasets used and/or analyzed during the current study are available from the corresponding author on reasonable request.

## Abbreviations

IOL: Intraocular lens
UDVA: Uncorrected distant visual acuity
UIVA: Uncorrected intermediate visual acuity
UNVA: Uncorrected near visual acuity
CDVA: Corrected distant visual acuity
CST: Contrast sensitivity
EDoF: Extended depth of focus
NEI VFQ: National Eye Institute Visual Functional Questionnaire
CPD: Cycles per degree
D: Diopters
SE: Spherical equivalent
logMAR: Logarithm of the minimal angle of resolution
